# Exploring the Link between Interoceptive Body Awareness and Suicidal Orientation in University Students: A Cross-Sectional Study

**DOI:** 10.3390/bs13110945

**Published:** 2023-11-17

**Authors:** Olga Lucia Montoya-Hurtado, Nicolás Gómez-Jaramillo, José María Criado-Gutiérrez, Jesús Pérez, Consuelo Sancho-Sánchez, Mercedes Sánchez-Barba, Clara Isabel Tejada-Garrido, Laura Criado-Pérez, Juan Luis Sánchez-González, Iván Santolalla-Arnedo, Pablo del Pozo-Herce, Raúl Juárez-Vela

**Affiliations:** 1 Research Department, Escuela Colombiana de Rehabilitación, Health and Sports Sciences, Bogota 110121, Colombia; olga.montoya@ecr.edu.co; 2 Doctoral Program in Program in Health, Disability, Dependency, and Well-Being, University of Salamanca, 37007 Salamanca, Spain; 3 Research Department, Universidad de Manizales, Engineering Program, Manizales 170003, Colombia; apuntalo@gmail.com; 4 Department of Physiology and Pharmacology, Faculty of Medicine, University of Salamanca, 37007 Salamanca, Spain; jmcriado@usal.es (J.M.C.-G.); sanchoc@usal.es (C.S.-S.); 5 Department of Medicine, Faculty of Medicine, University of Salamanca, 37007 Salamanca, Spain; jesusperez@usal.es; 6 Biomedical Research Institute of Salamanca (IBSAL), Prevention, and Early Intervention in Mental Health (PRINT), 26004 Logroño, Spain; raul.juarez@unirioja.es; 7 Department of Statistic, Faculty of Medicine, University of Salamanca, 37007 Salamanca, Spain; mersanbar@usal.es; 8 Department of Nursing, Faculty of Health Sciences, University of La Rioja, 26004 Logroño, Spain; ivansantolalla@unirioja.es; 9 Research Group in Care, GRUPAC, University of La Rioja, 26004 Logroño, Spain; 10 Department of Surgery, Faculty of Medicine, University of Salamanca, 37007 Salamanca, Spain; 11 Faculty of Nursing and Physiotherapy, Department of Nursing and Physiotherapy, Instituto de Investigación Biomédica de Salamanca (IBSAL), 37007 Salamanca, Spain; juanluisanchez@usal.es; 12 Department of Psychiatry, Fundación Jiménez Díaz University Hospital, 28040 Madrid, Spain; pablo.pozo@quironsalud.es; 13 Instituto de Investigación Sanitaria de la Fundación Jiménez Díaz, 28040 Madrid, Spain

**Keywords:** suicidal behavior, suicide, school and education, quantitative methodology, prevention

## Abstract

Introduction: The transition to college life can impact the mental health of students. There are mental health care strategies that promote connection with the body’s internal signals, which can help to improve mental well-being, manage emotions, and reduce the risk of suicide in university students. Aim: This study aimed to examine the association between interoceptive body awareness variables and suicidal orientation in a sample of 169 undergraduate students in Colombia. Methods: An observational, cross-sectional study was conducted in 2023 with Colombian students as the participants. Results: The findings revealed a significant and moderately negative correlation between the Multidimensional Assessment of Interoceptive Awareness (MAIA) total score and the Inventory of Suicide Orientation (ISO-30) total score (r = −0.54, *p* < 0.001). Confidence and self-regulation were identified as the most influential factors in the relationship between MAIA and ISO-30. Significant correlations were observed (*p* < 0.001), indicating moderate correlation values ranging from −0.43 to −0.57. Discussion: Our findings support the existence of a negative correlation between interoceptive body awareness and suicidal orientation. Further research is needed to better understand this relationship and to develop specific interventions based on body awareness to prevent suicide orientation. Conclusion: There are practical implications associated with recognizing the importance of body awareness in relation to decreasing suicidal orientation, and multidisciplinary teams addressing mental health can incorporate this knowledge.

## 1. Introduction

Suicide is the intentional act of taking one’s own life and usually progresses through several stages, including suicidal ideation (persistent thoughts of wanting to die), planning (developing a specific plan), preparation (taking steps to carry out the plan), the actual suicide attempt, and the outcome, which may be survival or death. Suicidal behavior can be initiated without the presence of an underlying mental disorder [1].

The World Health Organization (WHO) recognizes suicide as a public health problem. Suicide was the fourth leading cause of death in the 15–19-year-old age group in both males and females in the region of the Americas, with relatively similar numbers of deaths in this age group [2,3].

In the latest “Forensis” forensic medicine report for the year 2021 in Colombia, based on the distribution by age group, the highest number of suicides occurred in the 20–24-year-old age group, both in males and females. The highest suicide rate per 100,000 inhabitants was 9.24 in the 18–19-year-old age range [4].

When students enter higher education, they face unique challenges and pressures that can have a significant impact on their mental health [5]. The transition to college life involves new academic responsibilities, personal independence, and the need to adapt to diverse spiritual, ethnic, and gender contexts, as well as a different social environment [6].

In 2014, the report “Preventing Suicide: A Global Imperative” was published with the aim of raising awareness of the relevance of suicide and its prevention in public health programs. This report aims to encourage countries to develop comprehensive suicide prevention strategies within a multisectoral approach to public health. Suicide is also addressed in the Global Action Agenda for Mental Health and is an indicator of the Sustainable Development Goals, which aim to reduce premature mortality from noncommunicable diseases and promote mental health and well-being [7].

In this context, social determinants play an important role in the mental health of university students; external factors such as the socioeconomic environment, access to health services, the quality of education, and social support can influence students’ vulnerability and resilience to emotional challenges [8]. Positive social determinants, such as a healthy family environment and a strong social support network, improve students’ mental health and coping skills [8].

Some of the interventions that have shown advances in suicide prevention include suicide education, support networks, and interoceptive body awareness work [9,10]. Interoceptive awareness refers to the ability to perceive and understand internal body signals, such as visceral sensations, the heartbeat, breathing, and other bodily cues [11]. There is also the practice of mindfulness, which consists of intentionally and nonjudgmentally paying attention to the physical sensations, emotions, and thoughts of the present moment [12].

Through meditation, exercise, and mindful breathing, students can learn to connect with their bodies and respond more healthfully to the stresses and strains of college life [13,14]. These practices help cultivate self-awareness, emotional regulation, and resilience, which contributes to improved mental well-being and potentially reduced suicidal orientation [15].

Body awareness refers to a person’s ability to be aware of and connected to his or her body, physical sensations, and emotions. It involves paying attention to the signals sent by the body and responding appropriately to them [16]. By developing body awareness, individuals can improve their understanding of their own physical and emotional states, which can lead to improved self-regulation and overall well-being [16,17].

There are strategies using interoceptive body awareness that have demonstrated benefits for students’ mental health; for example, one study examined the effects of a telerehabilitation-based Basic Body Awareness Therapy (BBAT) approach on body awareness, musculoskeletal pain, sleep, and quality of life in college students during the COVID-19 pandemic. Two groups were evaluated: one received BBAT (*n* = 20) and the other served as a control group (*n* = 20). The treatment group received online BBAT three days a week for six weeks. The results showed significant improvements in the treatment group in terms of pain, sleep quality, body awareness, and quality of life. This telerehabilitation-based BBAT approach proved effective during the COVID-19 pandemic [18].

Another study evaluated the effects of Pilates training on body awareness and social appearance anxiety in university students. Eighty students participated, and they were divided into a Pilates group (*n* = 40) and a control group (*n* = 40). Significant differences were observed between the groups in terms of body awareness and social appearance anxiety scores after the Pilates program. These results highlight the importance of including Pilates programs in the training of physical therapy students and suggest the need for future research with larger numbers of participants [19].

Also, a study examined the relationship between mindfulness, well-being, and body satisfaction in university students. Data were collected from 369 students (220 females and 149 males) at Uşak University during the 2017–2018 academic year. The results showed that gender differences in body satisfaction were not significant. However, female students reported higher well-being, while male students showed higher conscious awareness. There was a positive, albeit weak, relationship between body awareness and well-being and body satisfaction. Both body satisfaction and well-being were significant predictors of mindfulness [20].

The importance of body awareness lies in its ability to help college students recognize and address stress, anxiety, and other emotional issues that may contribute to suicide risk. By cultivating a strong connection with their bodies, students can become more in tune with their emotions, physical sensations, and overall well-being. This heightened awareness allows them to identify early signs of distress and implement appropriate self-care and support strategies [17].

Emotional distress and psychological suffering can disconnect the person from their body, making it difficult to identify and manage signs of mental distress [16]. Studies have demonstrated the benefits of certain interventions [14,16,17]. However, no study has established the relationships between body awareness and suicidal orientation. The aim of this study was to analyze the relationship between body awareness variables and suicidal orientation in a population of undergraduate students in Colombia.

## 2. Methods

### 2.1. Study Design

An observational, cross-sectional study was conducted between February and April 2023. Convenience sampling was carried out with students studying Rehabilitation Sciences from a Colombian university. Undergraduate students were invited to participate on a voluntary basis, and no exclusion of any kind was made. The questionnaires were applied through Google Forms in three meetings, and a researcher was always present in case any student had any doubts.

This study was conducted with the approval of the bioethics committee of the Colombian School of Rehabilitation (ECR-CI-INV-121-2021) within the framework of the early warning program for mental health conditions, which is supported by the research unit and the university welfare unit. Students completed two online questionnaires in February 2023. The questionnaires used were the MAIA to measure body awareness and the ISO-30 to assess suicidal orientation. Before the students answered the questionnaires, the ethical considerations of the study were explained and informed consent was obtained.

### 2.2. Tools

In terms of the selection in relation to the questionnaires applied, it was considered that they were validated in a population of university students in Colombia and that they evaluated the variables under study. The questionnaires are described below.

#### 2.2.1. Multidimensional Assessment of Interoceptive Awareness (MAIA)

The Multidimensional Assessment of Interoceptive Awareness (MAIA) is a questionnaire used to measure interoceptive body awareness in individuals [21]. A study conducted in 2023 examined the psychometric properties of the 32-item MAIA in Colombian university students. The study reported an overall Cronbach’s alpha (α) of 0.90 and an omega coefficient (Ω) of 0.96. Additionally, the study proposed a seven-factor model, which, in terms of our application, was not considered due to the confidence intervals provided for the original version [11].

The 32-item MAIA consists of 8 dimensions that assess aspects of interoceptive awareness. It uses a Likert-type measurement scale ranging from 0 (never) to 5 (always). It provides a total score for the level of body awareness and a dimensional assessment. For the dimensional assessment, it is important to note that questions 5, 6, 7, 8, and 9 are reverse-scored. The MAIA total is given by the sum of all items divided by 32. The score is set on a scale of 0 to 5 for each dimension. The interpretation is that higher total scores indicate higher levels of positive awareness. Table 1 presents the definitions of the dimensions [22].

#### 2.2.2. Inventory of Suicide Orientation (ISO-30)

The ISO-30 questionnaire is composed of 30 questions that detect the possible presence of suicidal orientation [23]. It is self-administered, and the questions are formulated positively and negatively, with answers given on a four-position Likert-type scale (0: strongly disagree, 1: partially disagree, 2: partially agree, and 3: strongly agree).

The objective of the ISO-30 questionnaire is to assess the presence and intensity of suicidal orientation in an individual over a 30-day period. Its purpose is to assist in the early identification of individuals at risk of suicidal behaviors and to provide relevant information for intervention and treatment [24]. This questionnaire has been previously used in a Colombian population of university students with a Cronbach’s alpha of 0.899 [24]. For the present study, the Cronbach’s alpha was verified in university students, with a result of 0.9333 and a 95% confidence interval between 0.918 and 0.947.

The ISO-30 presents 5 dimensions. Scoring is on a scale between 0 and 18 for each dimension, between 0 and 90 for the total ISO-30, and either true or false for the critical factors [25]. Table 2 presents the definitions of the dimensions

### 2.3. Statistical Analysis

For the analysis of the data, we used the programming language Python. The normality of the variables analyzed was verified using the D’Agostino test; since, in some cases, the null hypothesis of the normality of the variables was rejected, it was decided to use the Spearman correlation, which is more robust for variables that do not correspond to the criteria of normality. In addition, considering that the ISO-30 critical factor variable is binomial, the biserial point correlation was applied in this case.

We examined whether there was a correlation and the level of significance of the correlation between the MAIA total score and the ISO-30 total score. When a significant correlation was found, an analysis of the relationships between all the dimensions of both questionnaires was performed.

## 3. Results

The questionnaires were distributed to 187 students, with 3 students opting not to participate. Out of the remaining 184 participants, 15 chose not to complete the ISO-30 questionnaire. The final sample included 169 students who provided responses to both questionnaires.

Among the students who completed the online questionnaires, 80% were female, 93% were single, and 86% were living in Bogotá. The age range of the participants spanned from 16 to 42 years, with an average age of 20 years.

Table 3 reveals varying degrees of variability among the specific dimensions of the MAIA. The “not distracted” and “attention regulation” dimensions showed greater variability, as evidenced by their standard deviations of 0.92 and 0.91, respectively. This suggests notable differences in how participants responded to these dimensions.

On the other hand, the “perception” and “emotional awareness” dimensions showed a more consistent level of response across participants, with standard deviations of 0.88 and 0.97, respectively. This implies that, in general, participants showed a higher level of interoceptive awareness in these areas.

The total ISO-30 scores indicate a considerable range of levels of suicidal orientation within the sample, with a standard deviation of 17.00 and a mean score of 34.00.

When specific dimensions of the ISO-30, such as low self-esteem, hopelessness, inability to cope, loneliness–isolation, and suicidal ideation, are examined, it becomes apparent that participants’ responses also varied significantly, with standard deviations ranging from 3.03 to 4.67.

It is important to note that higher scores on the ISO-30 dimensions correspond with a greater presence of suicidal orientation. Certain critical ISO-30 factors merit separate analysis, and these will be explored in greater detail in Section 3, along with the relationships identified with the MAIA dimensions.

It was found that there was a moderate and significant negative correlation between the total score of the MAIA and the total score of the ISO-30 (r = −0.54, *p* < 0.001).

Once the correlation between the MAIA and ISO-30 was established, the total score in the ISO-30 inventory was further analyzed in relation to the factors that compose the MAIA questionnaire. The findings are described below, as shown in Table 4.

There was a moderate and significant negative correlation between ISO-30 total and MAIA not distracting (r = −0.295, *p* < 0.001). A moderate and significant correlation was found between ISO-30 total and MAIA attention regulation (r = −0.400, *p* < 0.001). A moderate and significant correlation was found between ISO-30 total and MAIA self-regulation (r = −0.506, *p* < 0.001). A moderate and significant correlation was found between ISO-30 total and MAIA body listening (r = −0.419, *p* < 0.001). A moderate and significant correlation was found between ISO-30 total and MAIA trusting (r = −0.578, *p* < 0.001).

In addition, the study revealed that 15% of the population had critical factors. In examining the relationships with MAIA, Table 5 illustrates two weak but significant negative correlations between the ISO-30 critical factors and the MAIA factors of not distracted (r = −0.217, *p* < 0.001) and attention regulation (r = −0.209, *p* < 0.001); see Table 5.

Subsequently, all the factors of ISO-30 were analyzed with all of the factors of MAIA. Table 6 presents the findings.

According to Table 6, the most relevant relationships were found between the MAIA factors of trusting and self-regulation. All correlations were significant (*p* < 0.001) and generally moderate (correlation values ranging from −0.43 to −0.57). The correlations were only weak in the cases of self-regulation with ISO-30 low self-esteem (r = −0.38) and self-regulation with ISO-30 suicidal ideation (r = −0.38); see Table 6.

## 4. Discussion

The MAIA dimensions related to self-regulation and confidence in bodily sensations are significantly associated with critical factors of the ISO-30 questionnaire. Lower levels of self-regulation and confidence in bodily sensations are associated with a higher suicidal orientation and factors such as low self-esteem, hopelessness, inability to control emotions, and social isolation.

This may support the findings of a 6-month study involving 43 adults with recent suicidal ideation and non-suicidal self-injury (NSSI). Interoception assessments were conducted during an initial visit. Participants then completed biweekly assessments for 6 months, reporting the presence and severity/frequency of suicidal ideation and NSSI. The findings revealed that reduced body trust predicted the presence of suicidal ideation, the severity of suicidal ideation, and the presence of NSSI, while other metrics of interoceptive skills did not predict any outcomes or predicted only one or two outcomes [26]. This suggests that trusting one’s own body and feeling secure in it are essential components of one’s personality, and a lack of trust in the body can manifest as a negative attitude towards it [27].

In our study, correlations indicate that interoceptive awareness, particularly in the domains of not being distracted, attention regulation, self-regulation, body listening, and trust in bodily sensations, is associated with the presence of suicidal orientation. Individuals with a higher suicidal orientation tend to show lower levels of interoceptive awareness in these domains. However, it is important to note that these correlations do not imply causality, and further research may be needed to explore the underlying mechanisms of these relationships.

A study investigated whether attenuated interoceptive processing is associated with self-reported suicide attempts in individuals with a variety of psychiatric disorders including depression, anxiety, post-traumatic stress disorder, eating disorders, and/or substance use disorders. The results revealed that people who attempted suicide showed reduced responses to homeostatic threats to the body, such as an increased tolerance to sensations of breathlessness and an increased threshold to cold pain compared to those who did not attempt suicide. In addition, suicide attempters showed a lower accuracy in heartbeat perception and lower activity in the medial and posterior insula when paying attention to the sensations of the heart, an interoceptive organ vital for survival [28]. Taken together, these findings provide initial support for the hypothesis that an increased ability to engage in self-destructive and potentially life-threatening behaviors is associated, both behaviorally and neurobiologically, with reduced sensitivity to internal body cues [28].

Self-confidence and trust in others have been associated with greater psychological well-being and an improved quality of life; studies have found that confidence in interpersonal relationships and self-confidence are associated with a lower likelihood of experiencing depressive symptoms and greater psychological resilience [28]. This could justify the significant inverse correlation value in the MAIA confidence factor in relation to the ISO-30 factors. Importantly, the correlations were weakest for self-regulation with ISO-30 low self-esteem (r = −0.38) and self-regulation with ISO-30 suicidal ideation (r = −0.38). This suggests that the relationship between emotional self-regulation and these ISO-30-specific factors may be more complex and requires further analysis.

In our study, 15% of the student population was observed to have critical factors, which led to examining their association with the Multidimensional Assessment of Interoceptive Awareness (MAIA). The results presented in Table 5 reveal two weak but significant negative correlations between critical factors associated with suicidal orientation (ISO-30) and two MAIA factors: no distraction (r = −0.217, *p* < 0.001) and attention regulation (r = −0.209, *p* < 0.001) [29]. This is consistent with the results of a systematic review (2021) that drew on data from four databases and examined the influence of various measures of interoception along the suicide continuum. The analysis included 22 studies with a total of 14,988 participants. The findings of this review suggest that there is a relationship between interoceptive dysfunction and the risk of suicidal thoughts, intentions, and behaviors. Preliminary evidence was found indicating that interoceptive accuracy may be decreased in those who have previously attempted suicide. In addition, alterations in interoceptive sensitivity were identified at all stages of suicide, with individuals reporting distrust in their own bodily sensations and difficulties in maintaining and regulating attention to these sensations. This review highlights the importance of interoception in understanding suicidality but also underscores the need for further research addressing the causal relationships and mediating variables involved [30].

Studying the relationship between interoceptive body awareness and suicidal orientation is gaining interest, as evidenced by “Reconnecting to Internal Sensation and Experiences: A Pilot Feasibility Study of an Online Intervention to Improve Interoception and Reduce Suicidal Ideation” and “Body trust as a moderator of the association between exercise dependence and suicidality”; in addition to identifying inverse relationships of body trust and emotional awareness with suicidal intent, the authors of these papers recommend studies for generating scientific evidence [31,32].

## 5. Conclusions

Our findings support the existence of a negative correlation between body awareness and suicidal orientation. However, more research is needed to better understand this relationship and to develop specific body-awareness-based interventions to prevent suicidal risk.

This study examined the association between interoceptive body awareness and suicidal orientation in university students in Colombia. A significant and moderate correlation was found between the Multidimensional Assessment of Interoceptive Awareness (MAIA) total score and the Inventory of Suicide Orientation (ISO-30) total score (r = −0.54, *p* < 0.001). Confidence and self-regulation were identified as the most influential factors in the relationship between the MAIA and ISO-30. Significant correlations (*p* < 0.001) were observed with moderate correlation values ranging from −0.43 to −0.57.

Our findings support the existence of a negative correlation between interoceptive body awareness and suicidal orientation. Further research is needed to better understand this relationship and to develop specific interventions based on body awareness to prevent suicidal orientation.

In addition, other studies have suggested that self-confidence and trust in others are associated with greater psychological well-being and improved quality of life. This could justify the value of the significant inverse correlation in the confidence factor of the MAIA in relation to the ISO-30 factors. Importantly, the correlations were weakest for self-regulation with low self-esteem on the ISO-30 (r = −0.38) and self-regulation with suicidal ideation on the ISO-30 (r = −0.38). This suggests that the relationship between emotional self-regulation and these ISO-30-specific factors may be more complex and requires further analysis.

## 6. Limitations

A limitation of this study was the convenience sample, which was intended for initial screening. Future studies with larger, more diverse, and randomized samples are needed. The results presented here are based on a correlational study design, which cannot establish causality. Although the study identified a significant negative correlation between the MAIA total score and the ISO-30 total score, it is important to note that correlation does not imply causation. Future research using experimental or longitudinal designs could provide a more complete understanding of the causal relationships between these variables.

The sample was obtained from a university with professions that have a higher selection of women, which was a limitation for gender analysis.

## Figures and Tables

**Table 1:** MAIA: definitions of dimensions.

Dimension	Definition
Noticing	Measures the ability to maintain attention to interoceptive cues without being distracted by external factors
Not distracting	Refers to the ability to maintain focus on interoceptive signals without being distracted by external factors
Not worrying	Assesses the ability not to feel uncomfortable or anxious when experiencing internal bodily sensations.
Attention regulation	Refers to the ability to regulate and pay direct attention to internal bodily signals
Emotional awareness	Measures the ability to recognize and understand emotions and associated bodily sensations
Self-regulation	Assesses the ability to regulate and respond adaptively to internal body signals
Body listening	Refers to the ability to attend to and listen to the body’s needs and signals
Trusting	Measures confidence in one’s ability to interpret and understand internal body signals
MAIA Total	Sum of all scores

**Table 2:** ISO-30: definitions of dimensions.

Dimension	Definition
Low self-esteem	Negative and unhealthy perception that a person has of him/herself. It refers to a lack of confidence in one’s abilities, value, and self-image.
Hopelessness	This refers to an emotional state in which a person feels that there is no hope or possibility of improvement in his or her current situation or in the future.
Inability to control emotions	This means that a person has difficulty managing and modulating their emotions effectively.
Social isolation	This refers to a state in which an individual has limited contact or interaction with others. It often involves physical separation from social networks, such as friends, family, or the broader community. Social isolation can be voluntary, such as when someone chooses to live alone in a remote area, or it can be involuntary.
Suicidal ideation	Persistent thoughts of wanting to die
Total	Sum of all scores
Critical factors	True if the person scores two or more items (5, 10, 15, 20, 25, and 30) with a score higher than 2; false otherwise

**Table 3:** Characteristics of participants (*n* = 169).

Variable	Minimum	Maximum	Mean	Average	Standard Deviation
Age	16.00	47.00	20.00	21.60	5.22
MAIA Perceives	1.25	5.00	3.75	3.53	0.88
MAIA Not Distracted	0.00	4.33	2.00	1.93	0.92
MAIA Not Worrying	0.00	5.00	2.33	2.34	0.96
MAIA Attention Regulation	0.29	4.86	2.86	2.80	0.91
MAIA Emotional Awareness	0.80	5.00	4.00	3.76	0.97
MAIA Self-Regulation	0.00	5.00	2.75	2.69	1.18
MAIA Listening to the Body	0.25	4.75	2.50	2.50	1.09
MAIA Confidence	0.00	5.00	3.33	3.18	1.22
MAIA Total	0.70	4.02	2.90	2.84	0.59
ISO-30 Low Self-Esteem	0.00	18.00	6.00	6.67	3.75
ISO-30 Hopelessness	0.00	18.00	6.00	6.34	4.02
ISO-30 Inability to Control Emotions	0.00	17.00	9.00	9.08	3.03
ISO-30 Loneliness–Isolation	0.00	18.00	7.00	7.62	4.30
ISO-30 Suicidal Ideations	0.00	18.00	4.00	5.43	4.67
ISO-30 Total	3.00	81.00	34.00	35.14	17.00

**Table 4:** Correlation between ISO-30 total value and MAIA dimensions.

	x	y	r	Correlation Interp.	*p*-Unc	*p*-Value Interp.
0	ISO-30 Total	MAIA Noticing	0.026	Very weak	0.733	Not significant
1	ISO-30 Total	MAIA Not Distracting	−0.295	Weak	0.000	<0.001
2	ISO-30 Total	MAIA Not Worrying	−0.061	Very weak	0.428	Not significant
3	ISO-30 Total	MAIA Attention Regulation	−0.400	Moderate	0.000	<0.001
4	ISO-30 Total	MAIA Emotional Awareness	−0.116	Very weak	0.131	Not significant
5	ISO-30 Total	MAIA Self-Regulation	−0.506	Moderate	0.000	<0.001
6	ISO-30 Total	MAIA Body Listening	−0.419	Moderate	0.000	<0.001
7	ISO-30 Total	MAIA Trusting	−0.578	Moderate	0.000	<0.001

**Table 5:** Critical factors.

	x	y	r	Correlation Interp.	*p*-Unc	*p*-Value Interp.
0	ISO-30 Critical items	MAIA Noticing	0.080	Very weak	0.303	Not significant
1	ISO-30 Critical items	MAIA Not Distracting	−0.217	Weak	0.005	<0.01
2	ISO-30 Critical items	MAIA Not Worrying	−0.042	Very weak	0.586	Not significant
3	ISO-30 Critical items	MAIA Attention Regulation	−0.209	Weak	0.006	<0.01
4	ISO-30 Critical items	MAIA Emotional Awareness	−0.083	Very weak	0.283	Not significant
5	ISO-30 Critical items	MAIA Self-Regulation	−0.192	Very weak	0.012	<0.05
6	ISO-30 Critical items	MAIA Body Listening	−0.081	Very weak	0.296	Not significant
7	ISO-30 Critical items	MAIA Trusting	−0.169	Very weak	0.028	<0.05

**Table 6:** Correlation between relevant MAIA-ISO-30 factors.

	x	y	r	Correlation Interp.	*p*-Unc	*p*-Value Interp.
1	MAIA Self-Regulation	ISO-30 Low self-esteem	−0.380	Weak	0.0	<0.001
2	MAIA Self-Regulation	ISO-30 Hopelessness	−0.491	Moderate	0.0	<0.001
3	MAIA Self-Regulation	ISO-30 Inability to control emotions	−0.469	Moderate	0.0	<0.001
4	MAIA Self-Regulation	ISO-30 Social isolation	−0.493	Moderate	0.0	<0.001
5	MAIA Self-Regulation	ISO-30 Suicidal ideation	−0.376	Weak	0.0	<0.001
6	MAIA Trusting	ISO-30 Low self-esteem	−0.471	Moderate	0.0	<0.001
7	MAIA Trusting	ISO-30 Hopelessness	−0.531	Moderate	0.0	<0.001
8	MAIA Trusting	ISO-30 Inability to control emotions	−0.510	Moderate	0.0	<0.001
9	MAIA Trusting	ISO-30 Social isolation	−0.570	Moderate	0.0	<0.001
10	MAIA Trusting	ISO-30 Suicidal ideation	−0.432	moderate	0.0	<0.001

## Data Availability

Data are available on request from the first author.
